# RAGE has potential pathogenetic and prognostic value in nonintubated hospitalized patients with COVID-19

**DOI:** 10.1172/jci.insight.157499

**Published:** 2022-05-09

**Authors:** Katherine D. Wick, Lianne Siegel, James D. Neaton, Cathryn Oldmixon, Jens Lundgren, Robin L. Dewar, H. Clifford Lane, B. Taylor Thompson, Michael A. Matthay, on behalf of the ACTIV-3/TICO study group

**Affiliations:** 1 Cardiovascular Research Institute, University of California, San Francisco, San Francisco, California, USA.; 2 Division of Biostatistics and; 3 Division of Infectious Diseases, University of Minnesota, Minneapolis, Minnesota, USA.; 4 Division of Biostatistics, Massachusetts General Hospital, Boston, Massachusetts, USA.; 5 Centre of Excellence for Health, Immunity and Infections, Rigshospitalet, University of Copenhagen, Copenhagen, Denmark.; 6 Virus Isolation and Serology Laboratory, Applied and Developmental Research Directorate, Frederick National Laboratory, Frederick, Maryland, USA.; 7 Division of Clinical Research, National Institute of Allergy and Infectious Diseases (NIAID), NIH, Bethesda, Maryland, USA.; 8 Division of Pulmonary and Critical Care Medicine, Massachusetts General Hospital, Boston, Maryland, USA.; 9 Departments of Medicine and Anesthesia, University of California, San Francisco, San Francisco, California, USA.; 10 The ACTIV-3/TICO study group is detailed in Supplemental Acknowledgments.

**Keywords:** COVID-19, Pulmonology, Antigen, Clinical practice, Cytokines

## Abstract

**Background:**

The value of the soluble receptor for advanced glycation end-products (sRAGE) as a biomarker in COVID-19 is not well understood. We tested the association between plasma sRAGE and illness severity, viral burden, and clinical outcomes in hospitalized patients with COVID-19 who were not mechanically ventilated.

**Methods:**

Baseline sRAGE was measured among participants enrolled in the ACTIV-3/TICO trial of bamlanivimab for hospitalized patients with COVID-19. Spearman’s rank correlation was used to assess the relationship between sRAGE and other plasma biomarkers, including viral nucleocapsid antigen. Fine-Gray models adjusted for baseline supplemental oxygen requirement, antigen level, positive endogenous anti-nucleocapsid antibody response, sex, age, BMI, diabetes mellitus, renal impairment, corticosteroid treatment, and log_2_-transformed IL-6 level were used to assess the association between baseline sRAGE and time to sustained recovery. Cox regression adjusted for the same factors was used to assess the association between sRAGE and mortality.

**Results:**

Among 277 participants, baseline sRAGE was strongly correlated with viral plasma antigen concentration (ρ = 0.57). There was a weaker correlation between sRAGE and biomarkers of systemic inflammation, such as IL-6 (ρ = 0.36) and CRP (ρ = 0.20). Participants with plasma sRAGE in the highest quartile had a significantly lower rate of sustained recovery (adjusted recovery rate ratio, 0.64 [95% CI, 0.43–0.90]) and a higher unadjusted risk of death (HR, 4.70 [95% CI, 2.01–10.99]) compared with participants in the lower quartiles.

**Conclusion:**

Elevated plasma sRAGE in hospitalized, nonventilated patients with COVID-19 was an indicator of both clinical illness severity and plasma viral load. Plasma sRAGE in the highest quartile was associated with a lower likelihood of sustained recovery and higher unadjusted risk of death. These findings, which we believe to be novel, indicate that plasma sRAGE may be a promising biomarker for COVID-19 prognostication and clinical trial enrichment.

**Trial Registration:**

ClinicalTrials.gov NCT04501978.

**Funding:**

NIH (5T32GM008440-24, 18X107CF6, HHSN261201500003I, R35HL140026, and OT2HL156812).

## Introduction

COVID-19 causes a wide spectrum of clinical illness, from upper respiratory symptoms to severe respiratory failure and death. Several plasma biomarkers — such as IL-6, C-reactive protein (CRP), D-dimer, the neutrophil-to-lymphocyte ratio, and ferritin, among others — have been studied as markers of disease severity and prognosis (1–3). Many of the plasma biomarkers that have garnered high interest reflect general systemic inflammation, immune dysregulation, or coagulopathy and endothelial activation but are not specific to pulmonary epithelial injury. Furthermore, their relationship to viral burden and replication dynamics requires further study. Although the immune response is dysregulated in severe COVID-19 infection, COVID-19 pneumonia is characterized by relatively less systemic inflammation as compared with other causes of respiratory failure and the acute respiratory distress syndrome (ARDS; refs. 4–6). Autopsy studies demonstrate that the lungs are the primary site of viral involvement, even in cases of COVID-19 characterized by multiorgan damage (7, 8). However, it is unclear how pulmonary epithelial injury is related to viral burden, the host inflammatory response to infection, or both. A better understanding of the relationship among pulmonary epithelial injury, baseline disease severity, viral burden, immune response, and clinical outcomes might help identify those patients at highest risk of respiratory deterioration and potentially contribute to the identification of surrogate markers of treatment efficacy specific to pulmonary injury.

The receptor for advanced glycation end-products (RAGE) and its soluble form, sRAGE, are primarily expressed by type I pneumocytes (9, 10); they have been well-characterized as markers of pulmonary type I alveolar epithelial cell injury and as diagnostic and prognostic biomarkers in ARDS (11, 12). Although RAGE is implicated in a variety of both acute and chronic inflammatory processes outside the lung (13), elevated plasma sRAGE among patients with acute lung injury has greater specificity for pulmonary injury than other markers of systemic inflammation (14–16). The role of sRAGE in non-ARDS pneumonia, including COVID-19 pneumonia, has not been well-described. Limited prior studies have demonstrate an association between elevated plasma sRAGE and adverse outcomes among patients with COVID-19 pneumonia, but they have focused on short-term outcomes in cohorts outside of clinical trials without measurements of viral load or antibodies (17, 18).

The overall objective of this study was to measure plasma sRAGE in a well-characterized cohort of participants from a multicenter, prospective randomized controlled trial of the neutralizing monoclonal antibody bamlanivimab in hospitalized patients. In this trial, participants were randomized to either active monoclonal antibody treatment with bamlanivimab (7000 mg) or placebo treatment and were followed for 90 days for the primary outcome of sustained recovery (19). There was no treatment effect observed in the primary trial. These patients received high-level supportive care in addition to the study drug. Plasma viral nucleocapsid antigen levels and the presence of endogenous antibodies were measured, providing detailed information on viral burden and immune response that have not previously been studied in relation to sRAGE. The aims were to investigate (a) the association between baseline sRAGE and other baseline variables associated with COVID-19 severity, including viral antigen level, and (b) the relationship between baseline plasma sRAGE and the baseline oxygen requirement as well as clinical outcomes. We hypothesized that higher baseline sRAGE would be associated with a higher viral burden, a higher baseline oxygen requirement, and a lower likelihood of recovery at 90 days.

## Results

**Figure 1:**
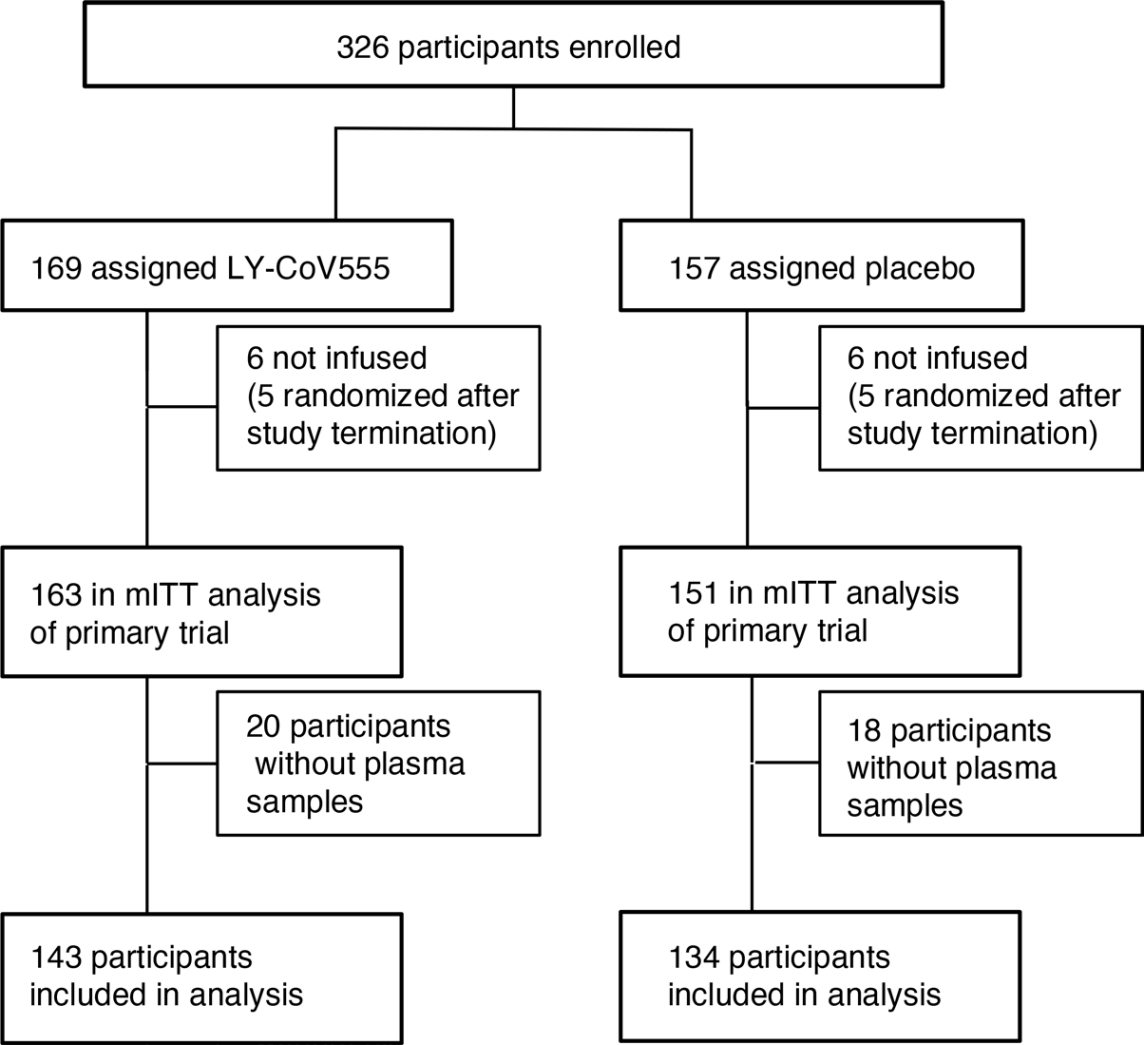
Study design.

Baseline sRAGE was measured for 277 of the 314 participants (88%) in the primary analysis of bamlanivimab (19), on the basis of whether plasma was available (Figure 1). There was no significant difference between patients with measured sRAGE and those without in terms of their baseline oxygen requirement (*P =* 0.73); symptom duration (*P =* 0.42); plasma SARS-CoV-2 antigen concentration (*P =* 0.37); study treatment arm (*P =* 0.86); corticosteroid treatment (*P =* 0.60); IL-6 (*P =* 0.40), D-dimer (*P =* 0.68), or CRP concentrations (*P =* 0.54). For participants without plasma available for sRAGE measurement, sRAGE values were considered to be missing completely at random and were not imputed. Plasma sRAGE concentrations skewed right (Supplemental Figure 1; supplemental material available online with this article; https://doi.org/10.1172/jci.insight.157499DS1). The median baseline sRAGE concentration was 3702 pg/mL (IQR, 2016–6807 pg/mL; range, 78–37,500 pg/mL).

### Cross-sectional baseline associations with sRAGE.

**Figure 2:**
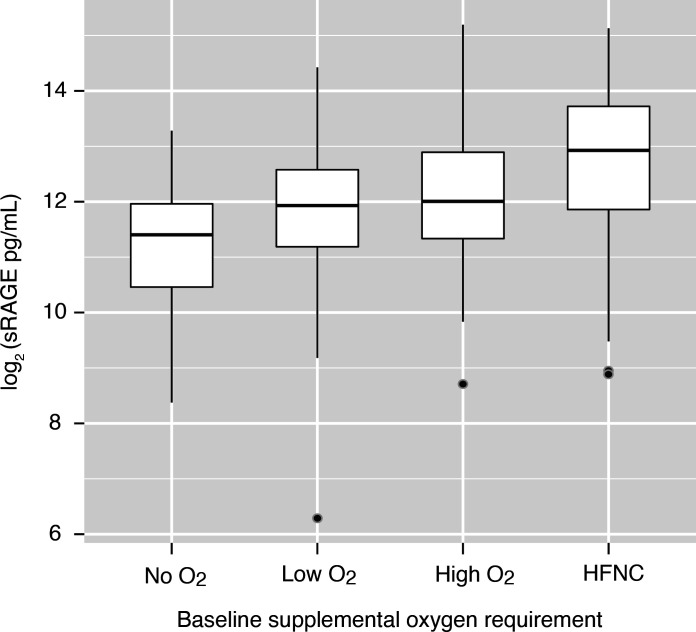
Log_2_-transformed plasma sRAGE concentration across levels of baseline oxygen requirement. Horizontal lines represent medians and boxes represent upper and lower quartiles. The bottom whiskers represent the lowest values within 1.5 IQR of the lower quartiles, and the top whiskers represent the highest values within 1.5 IQR of upper quartiles.

Participant characteristics by plasma sRAGE quartile are presented in Table 1. Baseline oxygen requirement differed significantly across sRAGE quartiles. In the lowest sRAGE quartile, 10% of participants required high flow nasal cannula oxygen (HFNC) or noninvasive ventilation as compared with 33% in the highest quartile. In contrast, 42% of participants in the lowest quartile required no supplemental oxygen, as compared with only 7% in the highest quartile. The distribution of plasma sRAGE by baseline oxygen requirement is depicted in Figure 2. Median plasma sRAGE did not differ significantly by symptom duration. Baseline viral antigen levels were highest among participants in the highest sRAGE quartile; the Spearman’s rank correlation between sRAGE and SARS-CoV-2 antigen level was 0.57 (*P <* 0.001, Supplemental Table 1). There was no significant difference in the percentage of participants with positive anti–SARS-CoV-2 nucleocapsid antibodies across quartiles of sRAGE (*P =* 0.43). IL-6, CRP, and PAI-1 differed significantly across quartiles of RAGE, whereas protein C did not (Table 1). The inflammatory markers IL-6 (ρ = 0.36) and CRP (ρ = 0.2) demonstrated a weaker correlation than sRAGE with viral antigen concentrations (Supplemental Table 1). Baseline biomarker concentrations were also compared across categories of baseline oxygen requirement (Supplemental Table 2). Protein C, PAI-1, and IL-6 did not differ significantly by baseline oxygen requirement, whereas CRP and D-dimer did. Median CRP in participants requiring no oxygen was 27 μg/mL (IQR, 14–56 μg/mL) versus 67 μg/mL (IQR, 36–90 μg/mL) in participants requiring HFNC or noninvasive ventilation (*P* < 0.001). Median D-dimer in participants requiring no oxygen was 822 ng/mL (IQR, 570–1418 ng/mL) versus 1273 ng/mL (IQR, 720–2204 ng/mL) ng/mL in participants requiring HFNC or noninvasive ventilation (*P =* 0.005).

### Predictors of sustained recovery.

**Figure 3:**
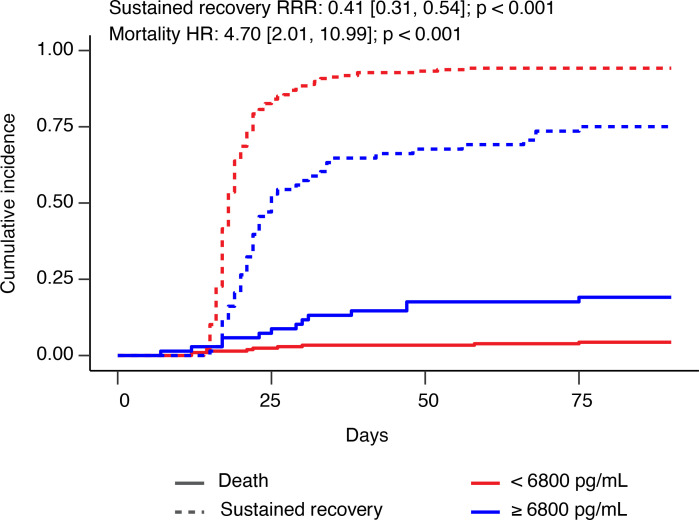
Cumulative incidence of sustained recovery and death stratified by sRAGE levels of equal to or more than 6800 pg/mL versus those less than 6800 pg/mL. Cumulative incidence of sustained recovery is represented by dashed lines, and death is represented by solid lines. The *P* value for sustained recovery is from Gray’s test. The *P* value for mortality is from the log-rank test from unadjusted Cox proportional hazards model.

Of the 277 participants with measured plasma sRAGE, 246 participants met the sustained recovery endpoint, 20 died before achieving sustained recovery, and 11 were right censored. Of the censored participants, 8 had not achieved the sustained recovery endpoint by day 90, while 3 were lost to follow-up. An additional 2 participants died later after achieving sustained recovery. In a model using continuous log_2_-transformed sRAGE, each doubling of plasma sRAGE was associated with an unadjusted recovery rate ratio (RRR) of 0.76 (95% CI, 0.69–0.84); however, this relationship was found to be nonlinear. When categorized by quartile, only the highest quartile of sRAGE was significantly associated with a lower rate of sustained recovery compared with the lowest quartile. Therefore, further analyses were categorized by sRAGE levels of equal to or more than 6800 pg/mL versus sRAGE levels of less than 6800 pg/mL. Among 207 participants with a baseline sRAGE level of less than 6800 pg/mL, 195 (94%) experienced sustained recovery within 90 days compared with 51 of 70 (73%) participants with baseline plasma sRAGE level equal to or more than 6800 pg/mL. The median time to recovery was 18 days (IQR, 17–21 days) in those with sRAGE levels of less than 6800 pg/mL and 22 days (IQR, 19–30 days) in those with sRAGE levels equal to or more than 6800 pg/mL (adjusted RRR, 0.64 [95% CI, 0.46–0.90], Figure 3 and Table 2).

The association between high plasma sRAGE and the rate of sustained recovery differed by baseline oxygen requirement. Among participants with no baseline oxygen requirement, there was no significant association between sRAGE levels equal to or more than 6800 pg/mL and recovery rate (unadjusted RRR, 0.87 [95% CI, 0.51–1.46] compared with sRAGE levels of less than 6800 pg/mL), although there were only 5 of 76 participants with sRAGE levels equal to or greater than 6800 pg/mL required no supplemental oxygen. Among 160 participants requiring supplemental oxygen, the RRR for participants with plasma sRAGE levels of 6800 pg/mL (*n =* 42, 26%) compared with those with plasma sRAGE levels of less than 6800 pg/mL was 0.56 (95% CI, 0.40–0.79). Among 41 participants requiring HFNC or noninvasive ventilation, the RRR for participants with plasma sRAGE levels equal to or more than 6800 pg/mL (*n =* 23, 56%) compared with those with plasma sRAGE levels of less than 6800 pg/mL was 0.33 (95% CI, 0.15–0.72). Baseline plasma sRAGE levels equal to or more than 6800 pg/mL were significantly associated with a worse 5-day pulmonary ordinal outcome in both adjusted and unadjusted models, as described in Supplemental Figure 2 and Supplemental Table 3.

### Association between sRAGE and mortality.

At 90 days, 20 patients had died. Unadjusted 90-day mortality differed significantly by plasma sRAGE category. At 90 days, 8 of 207 participants (3.9%) with baseline plasma sRAGE levels of less than 6800 pg/mL compared with 12 of 70 participants (17%) with sRAGE concentrations equal to or more than 6800 pg/mL had died. The HR for death for participants with baseline sRAGE concentrations equal to or more than 6800 pg/mL compared with participants with baseline sRAGE concentrations of less than 6800 pg/mL was 4.70 (95% CI, 2.01–10.99, *P <* 0.001). The association between plasma sRAGE levels equal to or more than 6800 pg/mL and mortality remained statistically significant when adjusted for baseline oxygen requirement (HR, 2.68, 95% CI, 1.05–6.81). The association was no longer statistically significant when adjusted for baseline oxygen requirement, clinical and demographic characteristics, viral antigen and endogenous anti-nucleocapsid antibody status, plasma IL-6 concentration, trial arm, and corticosteroid treatment (HR, 0.83; 95% CI, 0.24–2.95).

## Discussion

In this well-characterized multicenter cohort of hospitalized patients with COVID-19 from a prospective, randomized, double-blind trial, high baseline plasma sRAGE was associated with baseline severity of illness by both clinical (baseline oxygen requirement) and biologic criteria (viral antigen level and, to a lesser degree, markers of inflammation, especially IL-6). Our results indicate that plasma sRAGE is a biomarker that reflects both viral load and host response. The highest quartile of plasma sRAGE (≥6800 pg/mL) was strongly associated with higher baseline supplemental oxygen requirement and with a significantly lower likelihood of sustained recovery. We also found a significant association between plasma sRAGE and a worse 5-day pulmonary ordinal outcome. Therefore, sRAGE is a promising candidate biomarker for identifying those participants with COVID-19 pneumonia who are at greatest risk of worsening acutely and experiencing longer-term adverse outcomes. The association between high plasma sRAGE and 90-day sustained recovery retained significance after adjustment for other factors associated with illness severity, including the degree of systemic inflammation as represented by plasma IL-6 concentrations. Together these findings indicate that plasma sRAGE could play an important role in both biologic phenotyping and clinical risk stratification in future studies of COVID-19 pneumonia, including among patients who are not intubated.

The importance of sRAGE has been well established in ARDS studies as a marker of alveolar type I cell injury with functional implications for alveolar fluid clearance (11, 20) and predictive and prognostic significance (12, 21). However, the role of this biological marker in participants who were not mechanically ventilated has not been well studied. In this clinical trial cohort, baseline plasma sRAGE was highest among participants with the highest oxygen requirements at baseline. Thus, plasma sRAGE in nonintubated patients reflects a biological process (alveolar epithelial type I cell damage in response to viral infection) with the clear clinical corollary of increasing oxygen requirement. After adjustment for baseline oxygen requirement, there remained a strong and statistically significant independent association between baseline plasma sRAGE and 90-day sustained recovery in the entire sample. We also found that plasma sRAGE in the highest quartile was significantly associated with mortality in an unadjusted model and a model adjusted for baseline oxygen requirement but not in a fully adjusted model. It is important to note, however, that there were relatively few events (20 deaths) per the number of adjustment variables included in our model, which may have resulted in overfitting. The results of this study support the hypothesis that baseline plasma concentrations of sRAGE reflect direct pulmonary injury as a central pathophysiological process in COVID-19 pneumonia that is relevant to longer-term outcomes, possibly including mortality, and may be a meaningful prognostic biomarker in patients in whom severe respiratory failure has not yet developed.

Several prior studies have investigated the role of biomarkers of systemic inflammation and dysregulated coagulation in risk-stratifying participants with COVID-19 disease (1–3, 22). Although these biomarkers have value for understanding the host response to COVID-19, they are not specific to pulmonary injury. In this study, plasma biomarkers of inflammation and coagulation were less strongly correlated than sRAGE with plasma viral antigen concentrations, indicating that direct viral injury to the type I pneumocyte is likely a major contributor to both baseline oxygen requirement, short-term deterioration, and longer-term outcomes. IL-6 also differed significantly across sRAGE quartiles and was moderately correlated with sRAGE, reflecting that the host response to SARS-CoV-2 likely also contributes to alveolar epithelial damage, though perhaps to a lesser extent. Even among participants with no oxygen requirement at baseline, median sRAGE levels were comparable to those in cohorts of participants with ARDS (11, 20, 23). By contrast, median IL-6 and CRP levels of participants across all levels of disease severity were substantially lower than those observed in cohorts of participants with ARDS (5, 24). The striking elevation in plasma sRAGE concentrations by comparison indicates that (a) elevations in sRAGE are likely not substantially confounded by the contribution of systemic inflammation to the detection of this molecule, further underscoring their specificity to the pulmonary compartment, and (b) early pulmonary epithelial injury may be a sentinel event in severe disease. However, we cannot exclude the possibility that sRAGE elevations in this population are also reflective of some degree of systemic inflammation and that the increase in plasma sRAGE detected in baseline samples may precede changes in systemic inflammatory markers and markers of dysregulated coagulation.

The findings that sRAGE levels in hospitalized patients with COVID-19 are associated with plasma antigen levels, are moderately specific to pulmonary damage, and are associated with both short- and longer-term outcomes raise the question of whether plasma sRAGE could serve as a biomarker for predictive enrichment in clinical trials, as in a secondary analysis of the landmark trial of low tidal volume in ARDS (25), in which participants with higher baseline sRAGE were more likely to benefit from an low tidal volume strategy (26). Because this is, to our knowledge, one of the first investigations specifically analyzing the association between baseline plasma sRAGE and sustained recovery in COVID-19, we did not test an a priori threshold value. We found that sRAGE levels in the highest quartile had the strongest association with outcomes, a finding that should be externally validated in future studies before it can be prospectively applied for either predictive or prognostic enrichment.

Given its association with clinical outcomes and possible modification by treatment in previous observational studies (27), sRAGE may also be an appealing potential surrogate endpoint for further study of COVID-19 respiratory failure. In this study, we analyzed sRAGE collected at a single time point (baseline), but future directions include analyzing associations between changes in plasma sRAGE over time and short- and long-term outcomes, whether the rate of change is modified by treatment, and the development of point-of-care assays to rapidly measure sRAGE. Another intriguing possibility is targeting sRAGE directly through therapeutics. Although sRAGE has been identified as a potential causal intermediary in ARDS (28), further studies are required to clarify whether sRAGE in COVID-19 plays a role in amplifying lung injury as a damage-associated molecular pattern and could be directly targeted to mitigate pulmonary injury (29).

A major strength of this study is that the population comprised participants from multiple centers that were receiving a high level of standard care and were followed for 90 days. Measurement of viral antigen concentration and the presence of endogenous anti-nucleocapsid antibodies along with biomarkers of inflammation and coagulation provides potential additional novelty for this study. This study also has limitations. First, these findings may not be generalizable to populations outside of clinical trials. Second, we did not analyze the dynamics of sRAGE over time, and participants were not followed for outcomes beyond 90 days. Further studies of the association between plasma sRAGE both at baseline and over time and long-term outcomes, such as the development of postacute sequelae of SARS-CoV-2 infection, are needed. Third, not every patient from the primary study had plasma samples available for analysis. Because this was not related to clinical status or other participant-specific factors, however, the samples are considered to be missing completely at random, and the findings are likely generalizable to the entire sample. Finally, this study did not obtain samples directly from the distal air spaces, which may offer more information both about the effect of interventions and have differential associations with outcomes as compared with plasma samples (30). Among commonly studied ARDS biomarkers, however, sRAGE levels in the plasma are closely correlated with sRAGE levels in the airspaces, whereas the concentrations of other commonly studied biomarkers in ARDS that are derived from multiple organs are less well correlated between the compartments (30). Further, direct sampling of the airspaces in patients with COVID-19 has important biosafety limitations and is impractical in patients who are not yet endotracheally intubated.

In conclusion, plasma sRAGE is a promising pathogenetic and prognostic biomarker of alveolar epithelial injury in non-ARDS COVID-19 pneumonia. High baseline plasma sRAGE in COVID-19 pneumonia is associated with baseline severity of illness, antigen level, and both short-term deterioration and longer-term adverse outcomes. These potentially novel findings indicate that plasma sRAGE may be a promising biomarker in COVID-19 for both short-term and longer-term risk stratification in investigational treatments for COVID-19.

## Methods

Further information can be found in Supplemental Methods.

### Study design.

The methods and results for the ACTIV-3/TICO clinical trial of bamlanivimab (LY-CoV555, NCT04501978) have been previously reported (19). To summarize, patients 18 years or older were eligible for inclusion if they had a positive test for SARS-CoV-2 infection, with progressive symptoms suggestive of ongoing infection that required acute hospitalization. Detailed inclusion and exclusion criteria are provided in Supplemental Methods. Participants were excluded from the early phase of the trial if they had end-organ failure (vasopressor therapy; new renal replacement therapy; or invasive mechanical ventilation, extracorporeal membrane oxygenation, or mechanical circulatory support). Hospitalized patients were randomized to either active monoclonal antibody treatment with bamlanivimab (7000 mg) or placebo and followed for 90 days for the primary outcome of sustained recovery. Sustained recovery was defined as being discharged to home (or the same level of care the patient required prior to COVID-19 diagnosis) and remaining there for 14 consecutive days. Two ordinal outcomes, the pulmonary and “pulmonary plus” outcomes, were used for a prespecified early futility assessment when at least 300 participants were enrolled. A description of the criteria for the pulmonary and pulmonary-plus outcomes is provided in Supplemental Methods. The trial stopped early due to futility. The full trial protocol is available online with the primary trial results (19).

### Baseline biomarkers, antibody, and antigen measurements.

Blood samples were collected and centrifuged on the day of study enrollment. Serum and plasma samples were immediately cryopreserved at –80°C and stored in a central biospecimen repository for future analysis. Baseline plasma sRAGE, PAI-1, and protein C were measured using commercially available ELISA kits (sRAGE [Human RAGE Quantikine ELISA Kit] and PAI-1 [Human Serpin-E1/PAI-1 Quantikine ELISA Kit], R&D Systems; protein C [Protein C ELISA Kit], Helena Laboratories). All PAI-1 and protein C concentrations fell within the dynamic range of the assay. One PAI-1 measurement was excluded because of a high (>30%) coefficient of variation. Two plasma sRAGE concentrations fell above the dynamic range of the assay and were imputed by multiplying the highest value on the standard curve by 1.5 before correcting for the assay dilution factor. One plasma sRAGE concentration fell below the dynamic range of the assay and was assigned the lowest value on the standard curve. Plasma levels of IL-6 and serum levels of CRP were measured using electrochemiluminescence (Meso Scale Discovery). D-dimer was measured by an enzyme-linked fluorescent assay on a ViDAS instrument (bioMerieux). SARS-CoV-2 viral nucleocapsid protein levels were measured in plasma using a single-molecule immune bead assay (Quanterix). Antibodies (IgM, IgA, and IgG) against SARS-CoV-2 nucleocapsid were detected in plasma using ELISA (Platelia SARS-CoV-2 Total Ab Assay, Bio-Rad).

### Statistics.

The primary outcome of our study was sustained recovery through 90 days of follow-up, as in the primary trial. Baseline characteristics were compared across 4 groups of sRAGE corresponding to approximate quartiles. Cross-sectional comparisons of categorical variables were made with Fisher’s exact tests; continuous variables were compared across quartiles of sRAGE and across categories of baseline oxygen requirement using Kruskal-Wallis tests. Spearman’s rank correlations were calculated for each pair of biomarkers (sRAGE, protein C, PAI-1, IL-6, D-dimer, serum CRP, and antigen level) at baseline.

Both unadjusted models and models were used to analyze the association between plasma sRAGE and the primary outcome of 90-day sustained recovery using Fine-Gray models (accounting for the competing risk of death) or the secondary outcome of death using Cox proportional hazards models. The secondary outcome of 5-day ordinal oxygen requirement was also analyzed as described in Supplemental Methods. Adjustment variables were selected if they differed significantly by sRAGE quartile and are known to contribute to sRAGE production and clearance or known to contribute to COVID-19 outcomes, including mortality. They included baseline supplemental oxygen requirement (no oxygen, <4 L supplemental oxygen, ≥4 L supplemental oxygen but not high flow, or noninvasive ventilation/HFNC), trial treatment allocation, plasma nucleocapsid antigen concentration, endogenous anti-nucleocapsid antibody response, corticosteroid treatment, sex, age, BMI, diabetes mellitus, renal impairment, and log_2_-transformed IL-6 (pg/mL) level. This study represents the first investigation to our knowledge of sRAGE as a prognostic biomarker in COVID-19. Therefore, there was no a priori cutoff value selected for analysis, and plasma sRAGE was analyzed first as a log_2_-transformed continuous predictor to determine the increase in rate of each outcome associated with a doubling of sRAGE and then as a categorical predictor. Because the primary trial was negative and no significant interaction was observed between treatment assignment and the outcome of interest for any of the analyses described, both treatment groups were combined for all estimates. Statistical analyses were performed using R version 3.6.0. Fine-Gray and Cox proportional hazards models were fit using the “cmprsk” and “survival” packages, respectively. All *P* values are 2 sided, and a *P* value of less than 0.05 was considered statistically significant; no adjustment was made for multiple comparisons.

### Study approval.

Informed consent for study participation was obtained from either the participant or their authorized surrogate, and all study procedures were approved by a central institutional review board or ethics committee at each participating site (NIAID IRB no. 20-31756).

## Author contributions

KDW, LS, JDN, JL, BTT, and MAM contributed to the conception of the study and study design. KDW and LS prepared the first draft of the manuscript. KDW prepared the supplemental materials and tables and contributed to study design, biomarker measurements, and figure preparation. LS performed data analysis and contributed to figure preparation. JDN contributed to data analysis. RLD performed viral antigen measurements. JDN, CO, JL, RLD, HCL, BTT, and MAM contributed to the primary clinical trial and data acquisition. All authors critically reviewed the manuscript and provided revisions prior to submission.

## Supplementary Material

Supplemental data

ICMJE disclosure forms

## Figures and Tables

**Table 1:**
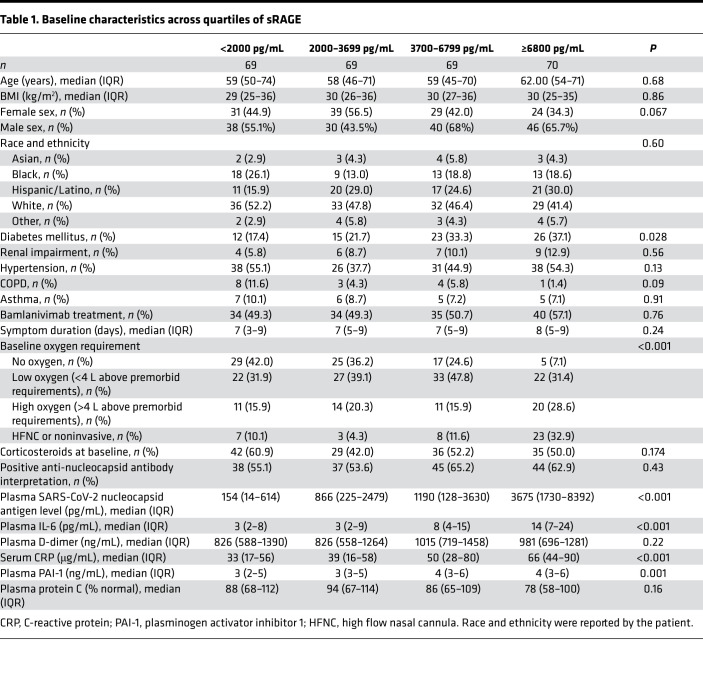
Baseline characteristics across quartiles of sRAGE

**Table 2:**
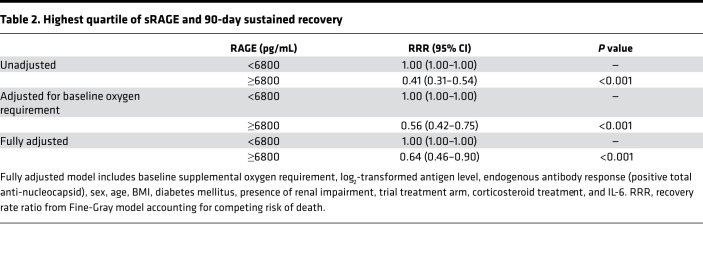
Highest quartile of sRAGE and 90-day sustained recovery

## Version 1 03/17/2022

In-Press Preview

## Version 2 05/09/2022

Electronic publication
