# Supplementary material for: RAGE has potential pathogenetic and prognostic value in nonintubated hospitalized patients with COVID-19
Source: JCI Insight. 2022 May 9;7(9):e157499. doi: 10.1172/jci.insight.157499 (PMC9090244; doi:10.1172/jci.insight.157499)
Supplement: Supplemental data [file jciinsight-7-157499-s133.pdf]

RAGE has potential pathogenetic and prognostic value in non-intubated hospitalized patients with COVID-19

Supplemental Material

TICO Study Group:

U.S. National Institute of Allergy and Infectious Diseases incl. Department of Clinical Research. H. Clifford Lane, *M.D.,* John Tierney, *B.Sc.N.*, Elizabeth S. Higgs, *M.D., D.M.T.H., M.I.A.,* Kevin Barrett, *R.N., B.Sc.N.,* Betsey R. Herpin, *M.Sc.N., C.C.R.C., R.N.,* Mary C. Smolskis, *B.Sc.N., M.A.,* Susan E. Vogel, *R.N., B.Sc.N.,* Laura A. McNay, *M.Sc.,* Kelly Cahill, *R.N., M.Sc., C.C.R.C., R.A.C.,* Page Crew, *Pharm.D., M.P.H., B.C.P.S.,* Matthew Kirchoff, *Pharm.D., M.Sc., M.B.A.,* Ratna Sardana, *B.A.,* Sharon Segal Raim, *M.P.H.*

Foundation for the National Institutes of Health, The Accelerating COVID-19 Therapeutic Interventions and Vaccines (ACTIV) and Operation Warp Speed. Stacey J. Adam, *Ph.D.,* Judy Currier, *M.D.*, Sarah Read, *M.D.,* Eric Hughes, *M.D., Ph.D.*, Rachel H. Harrigan, *M.D.*

INSIGHT SDMC, Division of Biostatistics, School of Public Health and School of Statistics, University of Minnesota, Minneapolis, MN, USA. James D. Neaton, *Ph.D.*, Laura Amos, Anita Carter, Gary Collins, *M.S.,* Bionca Davis, *M.P.H.,* Eileen Denning, *M.P.H.,* Alain DuChene, Nicole Engen, *M.S.,* Greg Grandits, *M.S.,* Birgit Grund, *Ph.D.,* Merrie Harrison, Nancy Hurlbut, Joseph Koopmeiners, *Ph.D.,* Gregg Larson, *M.A.,* Sue Meger, Shweta Sharma Mistry, *M.S.,* Thomas Murray, *Ph.D.,* Ray Nelson, *R.N.,* Kien Quan, *M.S.,* Siu Fun Quan, Cavan Reilly, *Ph.D.,* Greg Thompson, David Vock, *Ph.D.,* Deborah Wentworth, *M.P.H.*

Cardiothoracic Surgical Trials Network (CTSN) International Coordinating Center (ICC). Icahn School of Medicine at Mount Sinai, New York, USA. Annetine C. Gelijns, *Ph.D.*, Alan J. Moskowitz, *M.D.*, Emilia Bagiella, *Ph.D.*, Ellen Moquete, *R.N., B.Sc.N.*, Karen O'Sullivan, *M.P.H.*, Evan Accardi, *B.A.*, Emily Kinzel, *M.P.H.,* Gabriela Bedoya, Lopa Gupta*, M.P.H.,* Jessica R. Overbey, *Dr.P.H.*, Maria L. Padillia, *M.D.*, Milerva Santos, *M.P.A.*
CTSN Steering Committee Chair, Cleveland Clinic Foundation, Marc A. Gillinov, *M.D.*
U.S. National Heart Lung and Blood Institute. Marissa A. Miller, *D.V.M., M.P.H.*, Wendy C. Taddei-Peters, *Ph.D.,* Kathleen Fenton, *M.D., M.S.*

Duke University Hospital (71 participants enrolled). Peter K. Smith, *M.D.*, Christina E. Barkauskas, *M.D.*, Andrew M. Vekstein, *M.D.*, Emily R. Ko, *M.D.*, *Ph.D.*, Tatyana Der, *M.D.*, John Franzone, *M.D.*, Noel Ivey, *M.D.*, Thomas L. Holland, *M.D.*, Kathleen Lane, *B.Sc.N.*, *R.N.*, Andrew Bouffler, *B.Sc.*, Lauren M. McGowan, *B.Sc.*, *E.M.T.B.*, Ben Stallings, Jennifer Stout, *B.Sc.*, Whitney Franz, *B.Sc.*, R.N., Beth McLendon-Arvik, *Pharm.D.*, Beth A. Hollister, *B.Sc.N.*, *R.N.,* Dana M. Giangiacomo

Baylor, Scott and White Health (68 participants enrolled). Uriel Sandkovsky, *M.D.*, M.Sc., Robert L. Gottlieb, *M.D.*, *Ph.D.*, Michael Mack, *M.D.*, Mezgebe Berhe, *M.D.*, *M.P.H.*, Clinton Haley, M.*D.*, *M.P.H.*, Emma Dishner, *M.D.*, *M.P.H.*, Christopher Bettacchi, *M.D.*, Kevin Golden, *M.D.*, Erin Duhaime, *P.A.-C.*, Cedric Spak, *M.D.*, *M.P.H.*, Sarah Burris, Felecia Jones, Samantha Villa, Samantha Wang, Raven Robert, Tanquinisha Coleman, Laura Clariday, Rebecca Baker, Mariana

Hurutado, Nazia Iram, Michelle Fresnedo, Allyson Davis, Kiara Leonard, Noelia Ramierez, Jon Thammavong, Krizia Duque, Emma Turner, Aaron Killian, *Pharm.D.*, Adriana Palacious, *PharmD*, Edilia Solis, Janet Jerrow, Matthew Watts, Heather Whitacre, Elizabeth Cothran

<u>University of Southern California (10 participants enrolled).</u> Michael E. Bowdish, *M.D.*, *M.Sc.*, Zea Borok, *M.B., Ch.B.*, Noah Wald-Dickler, *M.D.*, Douglass Hutcheon, *M.D.*, Amytis Towfighi, *M.D.*, May Lee, *M.D.*, Meghan R. Lewis, *M.D.*, Brad Spellberg, *M.D.*, Linda Sher, *M.D.*, Aniket Sharma, *M.D.*, Anna P. Olds, *M.D.*, Chris Justino, *P.A.-C.,* Edward Lozano, *M.D.*, Chris Romero, *C.R.C.*, Janet Leong, *C.R.C.*, Valentina Rodina, *M.D.*, Christine Quesada, *C.R.C.*, Luke Hamilton, Jose Escobar

<u>Emory University (9 participants enrolled).</u> Brad Leshnower, *M.D.*, *F.A.C.S.*, William Bender, *M.D.*, *M.P.H.*, Milad Sharifpour, *M.D.*, *M.Sc.*, Jeffrey Miller, *M.D.*, Kim T. Baio, *R.N.*, *M.Sc.N.*, Mary K. McBride, *R.N.*, *B.Sc.N.*, *M.A.S.*, Michele Fielding, *R.N., B.Sc.N.*, *C.C.R.C.*, Sonya Mathewson, *R.N.*, *B.Sc.N.*, *C.C.R.C.*, Kristina Porte, *B.A.*, Missy Maton, *R.N.*, *B.Sc.N.*, Chari Ponder, *R.N.*, *B.Sc.N.*, Elizabeth Haley, *R.N.*, *B.Sc.N.*, *C.C.R.N.*, Christine Spainhour, *R.N.*, *C.C.R.C.*, Susan Rogers, *R.Ph.*, Derrick Tyler, *C.C.R.P.*

<u>University of Virginia Health Systems (1 participant enrolled).</u> Jeffrey M. Sturek, *M.D.*, *Ph.D.*, Andrew Barros, *M.D.*, *M.Sc.,* Kyle B. Enfield, *M.D.*, *F.C.C.M.*, *F.S.H.E.A.*, China J. Green, *B.Sc.*, *C.C.R.C.*, Rachel M. Simon, *R.N.*, *B.Sc.N.*, *C.C.R.C.*, Kara Thornton, *Pharm.D.*, *M.Ed.*, *C.C.R.P.*

<u>Ochsner Clinic.</u> Patrick E. Parrino, *M.D.*, *F.A.C.S.*, Stephen Spindel, *M.D.*, Aditya Bansal, *M.D.*, Katherine Baumgarten, *M.D.*, *F.A.C.P.*, *F.I.D.S.A.*, Jonathan Hand, *M.D.*, Derek Vonderhaar, *M.D.*,
Bobby Nossaman, *M.D.*, Sylvia Laudun, *D.N.P.*, *M.B.A.*, *R.N.*, *CPHQ*, DeAnna Ames, *M.Sc.*, Geralyn Isaac, *Pharm.D.*, Huan Dinh, *Pharm.D.*, Yiling Zheng, *Pharm.D.*, Hunter McDaniel, Nicolle Crovetto, *M.Sc.*, Abby Richardson

<u>West Virginia University.</u> Vinay Badhwar, *M.D.*, J. Awori Hayanga, *M.D.*, *M.P.H.*, Sunil Sharma, *M.D.*, Brian Peppers, *D.O.*, Paul McCarthy, *M.D.*, Troy Krupica, *M.D.*, Arif Sarwari, *M.D.*, *M.Sc.*, *M.B.A.*, Rebecca Reece, *M.D.*, Lisa Fornaresio, *Ph.D.*, Chad Glaze, *M.Sc.*, Raquel Evans, *B.Sc.N.*, *R.N.*, Fang Di, *R.N.*, *M.Sc.N.*, Shawn Carlson, *M.D.*, *M.Sc.*, Tanja Aucremanne, *B.Sc.N.*, *R.N.*, Connie Tennant, *B.Sc.N.*, *R.N.*, Lisa Giblin Sutton, *Pharm.D.*, Sabrina Buterbaugh, *Pharm.D.*, Roger Williams, *C.Ph.T.*, Robin Bunner, *B.Sc.*

<u>University of Maryland.</u> Ronson J. Madathil, *M.D.*, Joseph Rabin, *M.D.*, Andrea Levine, *M.D.*, Kapil Saharia, *M.D.*, Ali Tabatabai, *M.D.*, Christine Lau, *M.D.*, *M.B.A.*, James S. Gammie, *M.D.*, Maya-Loren Peguero, Kimberly McKernan, Matthew Audette, Emily Fleischmann, Kreshta Akbari, *M.Sc.*, Myounghee Lee, *Ph.D.*, *Pharm.D.*, Andrew Chi, *Pharm.D.*, Hanna Salehi, *Pharm.D.*, Alan Pariser, *Pharm.D.*, Phuong Tran Nyguyen, *Pharm.D.*, Jessica Moore, Adrienne Gee, Shelika

Vincent

Dartmouth-Hitchcock Medical Center. Richard A. Zuckerman, *M.D.*, *M.P.H.*, Alexander Iribarne, *M.D.*, *M.Sc.*, Sara Metzler, *B.Sc.N.*, *R.N.*, Samantha Shipman, *B.Sc.N.*, *R.N.*, Taylor Caccia, *B.A.*, Haley Johnson, Crystallee Newton, *B.A.*, *C.C.R.C.*, Doug Parr, *Pharm.D.*
Lutheran Medical Group. Vicente Rodriguez, *M.D.*, Gordon Bokhart, *Pharm.D.*, Sharon M. Eichman

Prevention and Early Treatment of Acute Lung Injury (PETAL) ICC. Massachusetts General Hospital, Boston, USA. B. Taylor Thompson *M.D.,* Crystal North *M.D.,* Cathryn Oldmixon *R.N.,* Nancy Ringwood *B.Sc.N*., Laura Fitzgerald *B.A./B.Sc.,* Ariela Muzikansky *R. N., B.A./B.Sc.,* Richard Morse *B.A./B.Sc.*
PETAL Steering Committee Chairperson: Johns Hopkins University. Roy G. Brower, *M.D*.
U.S. National Heart Lung and Blood Institute. Lora A. Reineck *M.D., M.S*., Neil R. Aggarwal, *M.D*.

ALIGNE Site Coordinating Center (SCC). Lead Investigators, Baystate Medical Center: Jay Steingrub, *M.D*.,
Brigham and Women's Hospital: Peter Hou *M.D*.
Baystate Medical Center: Jay S. Steingrub, *M.D.,* Mark A. Tidswell, *M.D.*, Lori-Ann Kozikowski, *R.N., B.Sc.N*., *C.C.R.N.,* Cynthia Kardos, *R.N., B.Sc.N*., C.C.R.N., Leslie De Souza

Boston SCC, Lead Investigators. Beth Israel Deaconess Medical Center: Daniel Talmor, *M.D.* and Nathan Shapiro, *M.D.*
University of Mississippi (2 participants enrolled): Alan E. Jones, *M.D.,* James Galbraith, *M.D.,* Utsav Nandi, *M.D.,* Rebekah Peacock, *R.N.*
Massachusetts General Hospital: Michael R. Filbin, *M.D., M.Sc.,* Kathryn Hibbert, *M.D.,* Blair Alden Parry, *C.C.R.A., B.A.,* Justin D, Margolin, *B.Sc.,* Kelsey Brait, *B.B.A., B.Sc.*

California SCC, Lead Investigators. University of California San Francisco: Michael A. Matthay, *M.D.*
David Geffen School of Medicine at UCLA: Gregory Hendey, *M.D.*
University of California San Francisco, University of San Francisco Mount Zion (4 participants enrolled): Michael A, Matthay, *M.D.,* Kirsten N. Kangelaris, *M.D.,* M.A.S, Kimberly J. Yee, *B.Sc.,* Alejandra E. Jauregui, *B.A.,* Hanjing Zhuo, *M.P.H.*
University of California Fresno (2 participants enrolled): Eyad Almasri, *M.D.*, Alyssa R. Hughes, *B.Sc.,* Rebekah L. Garcia, *C.C.R.P*., Kinsley A. Hubel, *M.D.*
Stanford University (1 participant enrolled): Angela J. Rogers, *M.D.,* Jennifer G. Wilson, *M.D., M.Sc.,* Rosemary Vojnik, *B.Sc.*, Jonasel Roque *B.Sc.*
University of Texas Health Science Center: Henry Wang, *M.D., M.Sc.,* Ryan M. Huebinger, *M.D.,* Bela Patel, *M.D.,* Elizabeth Vidales, *M.P.H., B.M.Sc.*

Colorado SCC, Lead Investigators. University of Colorado Hospital: Adit A. Ginde, *M.D., M.P.H,* Marc Moss, *M.D.*
University of Colorado Hospital (5 participants enrolled): Adit A. Ginde, *M.D., M.P.H.,* Amiran Baduashvili, *M.D.,* Lakshmi Chauhan, *M.D.,* David J. Douin, *M.D.,* Lani L. Finck, *B.A.,* Carrie Higgins, *R.N.,* Michelle Howell, *R.N.,* Jeffrey McKeehan, *M.Sc.N.,* Marc Moss, *M.D.*
National Jewish Health/St. Joseph Hospital: James H. Finigan, *M.D.,* William Janssen, *M.D.,* Peter Stubenrauch, *M.D.,* Christine Griesmer, *R.N., B.Sc.N., M.P.H.*

Michigan SCC, Lead Investigators: University of Michigan: Robert C. Hyzy, *M.D.,* Pauline K. Park, *M.D.*
University of Michigan (2 participants enrolled): Robert C. Hyzy, *M.D.,* Pauline K. Park, *M.D.,* Kristine Nelson, *R.N.,* Kelli McDonough, Jake I. McSparron, *M.D.,* Ivan N. Co, *M.D.,* Bonnie R. Wang, *M.D.,* Shijing Jia, *M.D.,* Barbara Sullins, Sinan Hanna, Norman Olbrich

Montefiore-Sinai SCC, Lead Investigators: Montefiore Medical Center: Michelle N. Gong, *M.D.,* Mount Sinai Hospital: Lynne D. Richardson, *M.D.*
Montefiore Medical Center Moses, Montefiore Medical Center Weiler: Michelle N. Gong, *M.D.,* M.S, Rahul Nair, *M.D.*
Banner University Medical Center Tucson: Jarrod M. Mosier, *M.D.,* Cameron Hypes, *M.D.,* Elizabeth Salvagio Campbell, *Ph.D.,* Billie Bixby, *M.D.,* Christian Bime, *M.D.,* Sairam Parthasarathy, *M.D.*

Ohio SCC, Lead Investigators: University of Cincinnati: R. Duncan Hite, *M.D.,* Ohio State University: Thomas E. Terndrup, *M.D*., Cleveland Clinic Foundation: Herbert P. Wiedemann, *M.D., M.B.A.*
Cleveland Clinic Foundation, Cleveland Clinic Fairview Hospital, Cleveland Clinic Marymount Hospital (2 participants enrolled): Abhijit Duggal, *M.D.,* Siddharth Dugar, *M.D.,* Debasis Sahoo, *M.D*., Kiran Ashok, *B.Sc.,* Alexander H. King, *M.Sc.,* Omar Mehkri, *M.D.*

Pacific Northwest SCC, Lead Investigators: Oregon Health and Science University: Catherine L. Hough, *M.D*.
University of Washington Medical Center: Bryce H. Robinson, *M.D.*
Harborview Medical Center, University of Washington Medical Center: Nicholas J. Johnson, *M.D.,* Bryce H. Robinson, *M.D.,* Stephanie J. Gundel, *R.D.,* Sarah C, Katsandres, *B.Sc.*
Oregon Health and Science University: Catherine L. Hough, *M.D.,* Akram Khan, *M.D*., Olivia F. Krol, Mistry Kinjal, Milad K. Jouzestani
Cedars-Sinai Medical Center: Peter Chen, *M.D*., Sam S. Torbati, *M.D.,* Yuri Matusov, *M.D*., June Choe, *M.D.,* Niree A. Hindoyan, *B.Sc.,* Susan E. Jackman, *B.Sc.N., M.Sc*., Emad Bayoumi, *M.B.A.,* Timothy Wynter, *B.Sc.,* Antonina Caudill, *M.P.H., C.P.H.,* Ethan Pascual, *M.A.,* Gregg J. Clapham, *M.A*., Lisa Herrera

Southeast SCC, Lead Investigators: Wake Forest Baptist Health: D. Clark Files *M.D.,* Chadwick Miller *M.D.*
Wake Forest Baptist Health (19 participants enrolled): D. Clark Files, *M.D.*, Keven W. Gibbs, *M.D.,* Lori S. Flores, *D.N.P.,* Mary E. LaRose, *R.N.*, *B.Sc.N,* Leigha D. Landreth, *R.N.*, *B.S.N*
University of Kentucky: Peter E. Morris, *M.D.,* Jamie L. Sturgill, *Ph.D.,* Evan P. Cassity, *M.Sc.,* Sanjay Dhar, *M.D.,* Ashley A. Montgomery-Yates, *M.D.,* Sara N. Pasha, *M.D.,* Kirby P. Mayer, *Ph.D.*
Virginia Commonwealth University: Marjolein de Wit, M.D., *M.Sc.,* Jessica Mason, *M.P.H*

Utah SCC, Lead Investigators: Intermountain Medical Center: Samuel M. Brown, *M.D.,* Joseph Bledsoe, *M.D.*
Intermountain Medical Center (23 participants enrolled): Kirk U. Knowlton, *M.D.,* Samuel Brown, *M.D.,* Michael Lanspa, *M.D.,* Lindsey Leither, *M.D.,* Ithan Pelton, *M.D.,* Brent P. Armbruster, *B.Sc.*, Quinn Montgomery, *B.Sc., AEMT*, Darrin Applegate, *B.Sc.,* Naresh Kumar, *M.P.H., C.C.R.P.,* Melissa Fergus, *B.Sc.,* Katie Brown, *B.Sc., R.N.,* Mardee Merrill, *B.Sc., C.C.R.P.,* Erna Serezlic, *B.Sc.,* Ghazal Palmer, *Pharm.D.,* Brandon Webb, *M.D.,* Valerie T. Aston, *M.B.A., R.R.T., C.C.R.P*.
University of Utah (5 participants enrolled): Estelle S. Harris, *M.D.,* Elizabeth A. Middleton, *M.D.,* Macy A.G. Barrios, *B.Sc.,* Jorden Greer, *B.Sc.,* Amber D. Schmidt, *B.Sc.,* Melissa K. Webb, *Pharm.D*., Robert Paine*, M.D.,* Sean J. Callahan, *M.D.*

Vanderbilt SCC, Lead Investigators: Vanderbilt University Medical Center: Wesley H. Self M.D., M.P.H., Todd W. Rice M.D., M.S.C.I.
Vanderbilt University Medical Center (23 participants enrolled): Wesley H. Self M.D., M.P.H., Todd W. Rice M.D., M.S.C.I., Jonathan D. Casey M.D., M.S.C.I., Jakea Johnson M.P.H, Christopher Gray R.N., Margaret Hays R.N., Megan Roth R.N.

INSIGHT Washington ICC, Veterans Affairs (VA) Medical Center, Washington DC: Virginia L. Kan, *M.D.*; Adriana Sánchez, *M.Sc.*; Laura Popielski, *M.P.H.*; Heather Rivasplata, *D.N..P*, *M.P.H.*; Melissa Turner, *M.S.W.*; Michael J. Vjecha, *M.D.*; Amy Weintrob, *M.D.*.
University of Texas Southwestern Medical Center, Dallas, TX (12 participants enrolled): Mamta K. Jain, *M.D.*, *M.P.H.*; Tianna Petersen, *M.Sc.*; Claudia Sanchez Lucas, *M.P.H.*.; Alexis Avery, *PS.M.*; Laura Hansen, *MA*; Natalie DellaValle, *Pharm.D.*, *B.C.P.S.*; Khanh-Hau Moss, *M.Sc.*, *PharmD*.

Hennepin Healthcare Research Institute, Minneapolis, MN (8 participants enrolled): Jason V. Baker, *M.D.*; Jonathan Klaphake, *B.Sc.*; Shari Mackedanz, *R.N.*; Rachael Goldsmith, *B.Sc.*; Hodan Jibrell, *B.Sc.*
Henry Ford Health System, Detroit, MI (5 participants enrolled): Norman Markowitz, *M.D.*, Erika Pastor, *R.N.*; Mayur Ramesh *M.D.*; Indira Brar, *M.D.*; Emanuel Rivers *M.D*.

Denver Public Health, Denver, CO (5 participants enrolled): Edward Gardner, *M.D.*; James Scott, *R.N.*; David Wyles, *M.D.*; Ivor Douglas, *M.D.*; Jason Haukoos, *M.D.*; Mitch Cohen, *M.D.*; Kevin Kamis, *M.P.H.*; Caitlin Robinson, *M.P.H*.
Georgetown University Medical Center, Washington, DC: Princy Kumar, *M.D.*, Maximilian Menna

VA Office of Research & Development: Victoria J. Davey, Ph.D., M.P.H.; VA Cooperative Studies Group (CSP) Site Coordinating Center (SCC): Kousick Biswas, *Ph.D.*; Cristin Harrington, *B.A*.
Michael E. DeBakey VA Medical Center, Houston, TX (4 participants enrolled): Barbara W. Trautner, *M.D.*, *Ph.D.;* Lavannya Pandit, *M.D.*, *M.Sc.*; Yigun Wang, *M.A*.
Miami Bruce Carter VA Health Care System, Miami, FL (3 participants enrolled): Paola Lichtenberger, *M.D.*; Gio Baracco, *M.D.*; Carol Ramos, *M.D.*; Lauren Bjork, *Pharm.D.*; Melyssa Sueiro, *M.Sc*.
San Francisco VA Health Care System, San Francisco, CA: Phyllis Tien, *M.D.*; Heather Freasier, *M.Sc.*, *R.D*.
Bay Pines VA Healthcare System, Bay Pines, FL: Theresa Buck, *M.D.*; Hafida Nekach, *M.D*.

INSIGHT Copenhagen ICC, CHIP (Centre of Excellence for Health, Immunity and Infections), Department of Infectious Diseases, Rigshospitalet, University of Copenhagen, Copenhagen, Denmark. Jens D. Lundgren, *M.D., PhD., D.M.Sc,* Dorthe Raben, *M.Sc.,* Daniel D. Murray, *Ph.D.,* Lars Peters, Bitten Aagaard, *B.Sc.N.,* Charlotte B. Nielsen, Katharina Krapp, *Ph.D.,* Bente Rosdahl Nykjær, Katja Lisa Kanne, *M.Sc. B.Sc.N*., Anne Louise Grevsen, *M.Sc. Dent.,* Zillah Maria Joensen, *B.Sc.N.,* Tina Bruun, *B.Sc.N.*

Denmark.

Copenhagen University Hospital, Amager Hvidovre, Center of Research & Disruption of Infectious Diseases, Department of Infectious Diseases (11 participants enrolled): Thomas Benfield, *M.D.,* Clara Lundetoft Clausen*, M.D.,* Nichlas Hovmand, *M.D.,* Simone Bastrup Israelsen, *M.D.,* Louise Krohn-Dehli, *R.N.,* Cæcilie Leding, *M.D.,* Dorthe Pedersen, *R.N.,* Karen Brorup Pedersen, *M.D.,* Louise Thorlacius-Ussing, *M.D.,* Michaela Tinggaard, *M.D.,* Sandra Tingsgård, *M.D.,* Signe Villadsen, *R.N.*
Herlev-Gentofte Hospital, Respiratory Medicine Section, Department of Internal Medicine (8 participants enrolled): Jens-Ulrik Stæhr Jensen, *M.D., Ph.D.,* Dorthe S. Høgsberg*, R.N.,* Christian P. Rønn, *M.D.,* Ema Rastoder, *M.D.,* Christian Søborg, *M.D., Ph.D.,* Christina Bergsøe, *B.Sc.,* Nuria M.S. Hissabu, *B.Sc.,* Bodil C. Arp, *B.Sc.*
Aarhus Universitetshospital, Skejby, Aarhus (5 participants enrolled). Lars Østergaard, *M.D., Ph.D. D.M.Sc*., Nina Breinholt Stærke, *M.D.*
Odense University Hospital, Department of Infectious Diseases (5 participants enrolled). Andreas Arnholdt Pedersen, *M.D.*, Inge K Holden, *M.D., Ph.D.*

Dept. of Infectious Diseases, Rigshospitalet, Copenhagen University Hospital (4 participants enrolled). Marie Helleberg, *M.D. Ph.D., D.M.Sc.,* Jan Gerstoft, *MD, D.M.Sc.*
North Zealand University Hospital, Department of Pulmonary and Infectious Diseases (4 participants enrolled). Tomas O. Jensen, *M.D.,* Birgitte Lindegaard, *M.D.*
Kolding Hospital, Department of Medicine (3 participants enrolled). Birgit Thorup Røge *MD. Ph.D*
Aalborg University Hospital, Department of Infectious Diseases. Henrik Nielsen, *M.D., D.M.Sc.*

Spain
INSIGHT SCC Spain, Hospital Universitari Germans Trias i Pujol, Badalona. Roger Paredes, *M.D., Ph.D.*, Anna Chamorro, *B.Sc.,* Ariadna Figuerola, *B.Sc.* Maria Exposito, *B.Sc.*
Hospital Universitari Germans Trias i Pujol, Badalona. Roger Paredes, *M.D., Ph.D.,* Lourdes Mateu, *M.D., Ph.D.,* Ana Martínez *B.Sc.,* Adrian Siles *B.Sc.*
Hospital General Universitario Gregorio Marañón, Madrid. Eduardo Fernández-Cruz, *M.D., Ph.D.,* Javier Carbone, *M.D., Ph.D.,* Paco López, *M.D.*
Barcelona Institute for Global Health (ISGlobal), Hospital Clínic - Universitat de Barcelona, Barcelona. José Muñoz, M.D., Ph.D., Daniel Camprubí, M.D. Almudena Legarda, B.VSc. Hospital Universitario La Paz, IdiPAZ, Madrid. Jose R Arribas, *M.D.*, Alberto Borobia, *M.D. Ph.D.,* Marta Mora- Rillo, *M.D. Ph.D.*, Irene García García, *M.D.*
Hospital Clínico San Carlos, Madrid. Vicente Estrada, *M.D., Ph.D.,* Noemi Cabello, *M.D.,* Maria Jose Nuñez, *M.D.*
Hospital del Mar, Barcelona. Juan P. Horcajada, *M.D., Ph.D.,* Elena Sendra, *M.D.,* Joan GómezJunyent, *M.D.*

INSIGHT Sydney ICC, The Kirby Institute, University of New South Wales, Sydney, Australia. Mark Polizzotto, *M.D., Ph.D.,* Gesalit Cabrera, *B.MSc., M.I.P.H,* Catherine Carey, *B.A., M.Sc.,* Christina C. Chang, *M.D., Ph.D.,* Sally Hough, *B.Sc.,* Sophie Virachit, *B.Sc., Ph.D.,* Amy Zhong, *B.Sc., M.P.H.*

Tan Tock Seng Hospital, NCID, Singapore (1 participant enrolled). *surname underlined, Barnaby E. Young, *M.D., Ph.D.,* Chia Po Ying, *M.D.,* Lee Tau Hong, *M.D.,* Ray J. Lin, *M.D.* , David Lye, *M.D.,* Sean Ong, *M.D.,* M.Med., Puah Ser Hon, *M.D.,* Yeo Tsin Wen, *M.D., Ph.*, Diong Shiau Hui B.Bio, *M.Sc., M.M.Sc.,* Juwinda Ongko, *B.Sc.*

INSIGHT London ICC, MRC Clinical Trials Unit at UC, London, UK. Abdel G. Babiker, *Ph.D.,* Sarah L. Pett, *M.D.,* Fleur Hudson, *B.Sc.*, Mahesh KB Parmar, *Ph.D*, Anna Goodman, *M.D., Ph.D.,* Jonathan Badrock, *B.Sc.,* Adam Gregory, *M.A.*

Greece SCC, National & Kapodistrian University of Athens Medical School. Giota Touloumi, *Ph.D.,* Vicky Gioukari, *B.Sc.*

Uganda SCC, JCRC/MRC/UVRI Uganda Research Unit. Joseph Lutaakome, *M.D.,* Cissy M. Kityo, *M.D*., Francis Kiweewa, *M.D.*

Eli Lilly and Company, Indianapolis, IN, USA. Paul Klekotka, M.D., Ph.D., Karen Price, Ph.D., Ajay Nirula, M.D., Ph.D.

Gilead Sciences, Foster City, CA, USA. Anu Osinusi, M.D. M.P.H., Huyen Cao, M.D.

Leidos Biomedical Research, Inc., Frederick, MD, USA. R. Baseler, *M.Sc.*, Marc J. Teitelbaum, *M.D., M.Sc.*, Shelly M. Simpson, *M.Sc.*, Molly J. Buehn, *M.Sc.*, David Vallée, *Pharm.D., M.P.H*., Vanessa Eccard-Koons, *M.Sc.*, Stacey Kopka, *M.Sc.*, Theresa M. Engel, *M.F.S.*, Cynthia Osborne, *B.Sc.*, Leah Giambarresi MacDonald, *M.Sc., R.A.C*., Liam M. Harmon, *C.C.R.A.,* Denise M. Shelley, *M.Sc.,* Mi Ha Kim, Ph.D., *C.C.R.P.*, Joy Beeler-Knights, *M.P.H., C.C.R.A., A.C.R.P.-C.P.M.*, Lindsey Yeon, Robin L. Dewar, *Ph.D.,* Helene C. Highbarger, *M.Sc.,* Perrine Lallemand, *B.S.*

Advanced Biomedical Laboratories, LLC., Cinnaminson, NJ, USA. Amanda Kubernac, Bhakti Patel, Kenneth Smith, Robert Kubernac, Norman P. Gerry, *Ph.D.*, Marie L. Hoover, *Ph.D.* PCI Pharma Services. Craig Brown, Nadine DuChateau, Adam Flosi, Les Johnson, Amy Treagus, and Christine Wenner.

Supplemental Methods:

Inclusion and exclusion criteria

Patients were eligible for the ACTIV-3/TICO LY-CoV555 trial if they were □18 years of age, had a positive PCR or other nucleic acid test for SARS-CoV-2 more than 3 days prior to randomization and progressive symptoms suggestive of ongoing infection, symptom duration □ 12 days, and required hospitalization for COVID-19 and NOT for public health or quarantine purposes. Subjects were excluded if they: had previously received any SARS-CoV-2 hyperimmune intravenous immunoglobulin, convalescent plasma from person who recovered from COVID-19, or any SARS-CoV-2 monoclonal antibody prior to hospitalization; were unwilling to forego other COVID-19 treatment trials until after day 5; had a condition for which, in the opinion of the responsible investigator, would not be in the best interest of the patient or would limit protocol-specific assessments; were expected to be unable to participate in study procedures; or were of child-bearing potential or a partner of someone of childbearing potential and were unable or unwilling to either abstain from sexual intercourse or use appropriate contraception. Subjects were also excluded if any of the following were present at enrollment (stage 1 only):

- stroke
- meningitis
- encephalitis
- myelitis
- myocardial infarction
- myocarditis
- pericarditis
- symptomatic congestive heart failure (NYHA class III-IV) i. arterial or deep venous thrombosis or pulmonary embolism

Or if there were current or imminent requirement for any of the following (stage 1 only):

- invasive mechanical ventilation
- ECMO
- mechanical circulatory support
- vasopressor therapy
- commencement of renal replacement therapy at this admission (i.e. not patients on chronic renal replacement therapy).

Outcomes

Day 5 Ordinal Outcomes

The 2 ordinal outcomes are assessed at day 5. The first ordinal outcome is a 7-category outcome largely based on oxygen requirements. The highest category that applies on day 5 was assigned. This outcome is referred to as the "pulmonary" ordinal outcome and is defined below:

1. Can independently undertake usual activities with minimal or no symptoms
2. Symptomatic and currently unable to independently undertake usual activities but no need of supplemental oxygen (or not above premorbid requirements)
3. Supplemental oxygen (<4 liters/min, or <4 liters/min above premorbid requirements)
4. Supplemental oxygen (≥4 liters/min, or ≥4 liters/min above premorbid requirements, but not high-flow oxygen)
5. Non-invasive ventilation or high-flow oxygen
6. Invasive ventilation, extracorporeal membrane oxygenation (ECMO), mechanical circulatory support, or new receipt of renal replacement therapy
7. Death

The second ordinal outcome, also assessed at Day 5, captures the range of organ dysfunction that may be associated with progression of Coronavirus-Induced Disease 2019

(COVID-19), such as respiratory dysfunction and coagulation-related complications. Again, the highest category that applies on day 5 was assigned. This outcome is referred to as the "pulmonary+" ordinal outcome. The 7 categories of the pulmonary+ ordinal outcome assessed at Day 5 are:

1. Can independently undertake usual activities with minimal or no symptoms
2. Symptomatic and currently unable to independently undertake usual activities but no need of supplemental oxygen (or not above premorbid requirements)
3. Supplemental oxygen (<4 liters/min, or <4 liters/min above premorbid requirements)
4. Supplemental oxygen (≥4 liters/min, or ≥4 liters/min above premorbid requirements, but not high-flow oxygen) or any of the following: stroke (NIH Stroke Scale [NIHSS] ≤14), meningitis, encephalitis, myelitis, myocardial infarction, myocarditis, pericarditis, new onset CHF NYHA class III or IV or worsening to class III or IV, arterial or deep venous thromboembolic events.
5. Non-invasive ventilation or high-flow oxygen, or signs and symptoms of an acute stroke (NIHSS >14)
6. Invasive ventilation, ECMO, mechanical circulatory support, vasopressor therapy, or new receipt of renal replacementtherapy
7. Death

The present study focuses on the pulmonary outcome since the results were similar across the two ordinal outcome definitions.

<u>Time from randomization to sustained recovery:</u>

The primary endpoint of the ACTIV-3/TICO LY-CoV555 trial was time from randomization to sustained recovery, where sustained recovery is defined as being discharged from the index hospitalization, followed by being alive and *home* for 14 consecutive days prior to Day 90. *Home* is defined as the level of residence or facility where the participant was residing prior to hospital admission leading to enrollment in this trial (the index hospitalization).

Residence or facility groupings to define home are:

1) **Independent/community dwelling** with or without help, including house, apartment, undomiciled/homeless, shelter, or hotel;

2) **Residential care facility** (e.g., assisted living facility, group home, other non-medical institutional setting); 3) **Other healthcare facility** (e.g., skilled nursing facility, acute rehab facility); and

4) L**ong-term acute care hospital** (hospital aimed at providing intensive, longer term acute care services, often for more than 28 days).

Lower (less intensive) level of residence or facility will also be considered as home. By definition, “home” cannot be a “short-term acute care” facility. Participants previously affiliated with a “long-term acute care” hospital recover when they return to the same or lower level of care.

Readmission from “home” may occur and if this occurs within 14 days of the first discharge to “home”, then the primary endpoint will not be reached until such time as the participant has been at home for 14 consecutive days. Participants residing in a facility solely for public health or quarantine purposes will be considered as residing in the lowest level of required residence had these public health measures not been instated.

Supplemental statistical methods:

Unadjusted and adjusted proportional odds models (adjusted for the same covariates as in the Fine-Gray and Cox proportional hazards models) were used to analyze the association between plasma sRAGE and the 5-day ordinal pulmonary outcome. The proportional odds models were parameterized so that a summary odds ratio (OR) greater than 1 corresponded to better outcomes in the given group. Proportional odds models were fit using the “MASS” package in R version 3.6.0.

Supplemental Results:

Predictors of Day 5 Ordinal Outcome

We tested the association between baseline sRAGE concentration and the 5-day ordinal pulmonary outcome. Based on the model using $\log_2$-transformed continuous sRAGE as a predictor, each doubling in plasma sRAGE was associated with an unadjusted OR of 0.49 (95% CI 0.41-0.59) and a fully adjusted OR of 0.64 (95% CI 0.56-0.87) for favorable pulmonary outcome. However, the association between 5-day pulmonary outcome and baseline sRAGE quartile did not follow a linear pattern, with only the highest quartile of sRAGE demonstrating a significant association with 5-day pulmonary outcome when compared with the lowest quartile. Therefore, we next tested the association between a baseline sRAGE ≥ 6800 pg/mL and the 5day pulmonary ordinal outcome **(Supplemental 3)**. A baseline sRAGE concentration of 6800 pg/mL or higher was associated with an unadjusted OR for favorable pulmonary outcome of 0.10 (95% CI 0.06-0.18) as compared with the lowest three quartiles. The odds of favorable pulmonary outcome remained significantly lower among participants with plasma

sRAGE ≥ 6800 pg/mL after adjustment for baseline clinical and demographic characteristics, baseline supplemental oxygen requirement, viral antigen and antibody status, corticosteroid treatment, and plasma IL-6 concentration (OR 0.22, 95% CI 0.11-0.43). Five-day ordinal pulmonary outcomes by quartile of sRAGE for the combined treatment groups are shown in **Supplemental**

Figure 2.

Because there was a significant interaction between baseline pulmonary status and dichotomous baseline sRAGE concentration in an unadjusted model for 5-day ordinal pulmonary outcome ($p<0.001$), we estimated the association between having plasma sRAGE concentration in the highest quartile and the 5-day pulmonary outcome within the following subgroups: participants requiring no supplemental oxygen (n = 76), those requiring supplemental oxygen but not HFNC/non-invasive ventilation (n = 160), and those requiring HFNC/non-invasive ventilation (n = 41). Only 5 participants with no baseline oxygen requirement had a plasma sRAGE above 6800 pg/mL, and there was no significant association between plasma sRAGE ≥6800 pg/mL and 5-day pulmonary outcome among these participants (OR 0.35, 95% CI 0.05-2.52). Among those requiring simple nasal cannula oxygen at any level, the summary odds ratio for more favorable pulmonary outcome at 5 days was 0.23 (95% CI 0.12 – 0.45) for participants with plasma sRAGE ≥6800 pg/mL compared to <6800 pg/mL. Among participants requiring HFNC or non-invasive ventilation, those with a plasma sRAGE level ≥6800 pg/mL had odds of a more favorable outcome 0.04 times those with lower plasma sRAGE levels (95% CI 0.01-0.19).

Supplemental Table 1. Spearman rank correlations between baseline biomarkers

	Protein C (% normal)	PAI-1 (ng/mL)	sRAGE (pg/mL)	IL-6 (pg/mL)	Ddimer	CRP (ug/mL)	Antigen (pg/mL)
**Protein C (% Norm)**	1	0.05	-0.14	-0.26	-0.21	-0.17	-0.15
**PAI-1 (ng/mL)**	0.05	1	0.24	0.11	-0.02	0.12	0.06
**RAGE (pg/mL)**	-0.14	0.24	1	0.41	0.1	0.31	**0.57**
**IL-6 (pg/mL)**	-0.26	0.11	0.41	1	0.34	0.35	0.36
**D-dimer (ng/mL)**	-0.21	-0.02	0.1	0.34	1	0.35	-0.04
**CRP (ug/mL)**	-0.17	0.12	0.31	0.35	0.35	1	0.2
**Antigen (pg/mL)**	-0.15	0.06	0.57	0.36	-0.04	0.2	1

Supplemental Table 2. Baseline plasma biomarker concentrations by baseline supplemental oxygen requirement

	No oxygen	Low oxygen (□4 liters)	High oxygen (>4 liters)	High flow or non-invasive ventilation	p
**sRAGE category (%)**					<0.001
**< 2000 pg/mL**	29 (38.2)	22 (21.2)	11 (19.6)	7 (17.1)	
**2000-3699 pg/mL**	25 (32.9)	27 (26.0)	14 (25.0)	3 (7.3)	
**3700-6799 pg/mL**	17 (22.4)	33 (31.7)	11 (19.6)	8 (19.5)	
**≥ 6800 pg/mL**	5 (6.6)	22 (21.2)	20 (35.7)	23 (56.1)	
**Positive anti-nucleocapsid antibody interpretation (%)**	30 (39.5)	68 (65.4)	40 (71.4)	26 (63.4)	0.001
**Viral antigen (pg/mL) (median [IQR])**	800 [102, 1980]	1188 [203, 4290]	860 [98, 3110]	1861 [181, 4940]	0.084
**IL-6 (pg/mL) (median [IQR])**	8 [3, 15]	5 [2, 11]	6 [3, 18]	8 [3, 19]	0.27
**D-dimer (ng/mL) (median [IQR])**	822 [570, 1418]	831 [609, 1211]	1001 [800, 1288]	1273 [719, 2204]	0.005
**CRP (ug/mL) (median [IQR])**	27.83 [14, 56]	44 [26, 77]	56 [39, 95]	67 [36, 90]	<0.001
**PAI-1 (ng/mL) (median [IQR])**	4 [2, 5]	4 [3, 6]	3 [2, 6]	4 [3, 6]	0.39
**Protein C (% normal) (median [IQR])**	85 [62, 107.35]	90 [70, 111]	84 [62, 110]	84 [58, 112]	0.45

Supplemental Table 3. Association between plasma sRAGE and 5-day ordinal pulmonary outcome

	**Per Quartile (unadjusted)**		
	**sRAGE (pg/mL)**	**OR [95% CI]**	**p-value**
	< 2000	1.00 [1.00, 1.00]	-----
	2000-3699	1.43 [0.79, 2.60]	0.244
	3700-6799	0.62 [0.34, 1.13]	0.117
	≥6800	0.10 [0.05, 0.18]	< 0.001
	**Highest Quartile (6800 pg/mL) vs. Lower 3 Quartiles**		
	**sRAGE (pg/mL)**	**Summary OR [95% CI]**	
**Unadjusted**	< 6800	1.00 [1.00, 1.00]	-----
	6800	0.10 [0.06, 0.18]	< 0.001
**Adjusted for baseline oxygen requirement**	< 6800	1.00 [1.00, 1.00]	-----
	≥6800	0.18 [0.10, 0.32]	< 0.001
**Fully adjusted**	< 6800	1.00 [1.00, 1.00]	-----
	≥ 6800	0.22 [0.11, 0.43]	< 0.001
	**Continuous (unadjusted)**		
**$\log_2$(RAGE)**		0.49 [0.41, 0.59]	<0.001

Fully adjusted model includes baseline supplemental oxygen requirement, $\log_2$-transformed antigen level, endogenous antibody response (positive total anti-nucleocapsid), gender, age, body mass index, diabetes mellitus, presence of renal impairment, trial treatment allocation, corticosteroid treatment, and interleukin-6.

Supplemental Figures

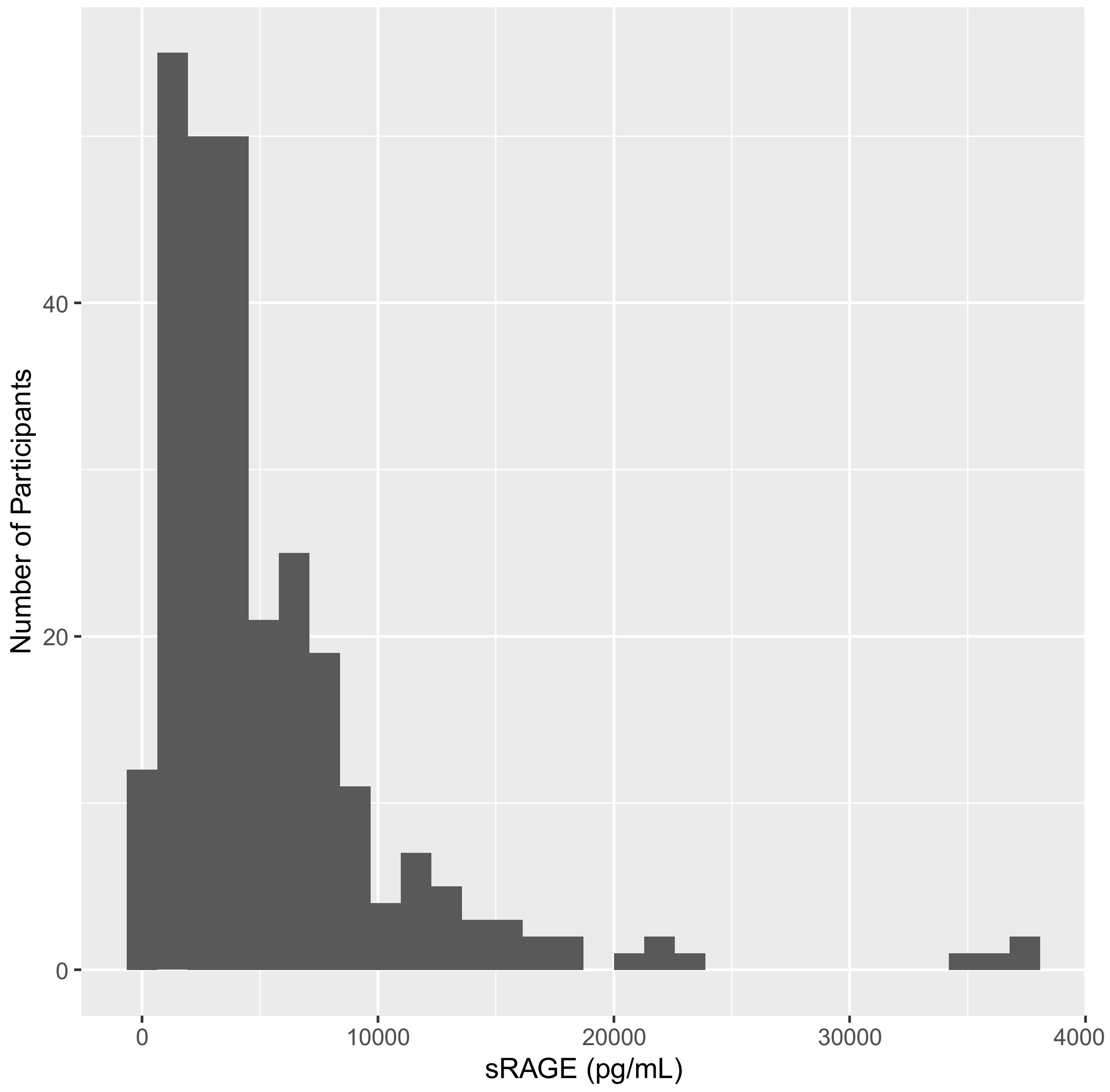

Supplemental Figure 1. Distribution of plasma sRAGE in 277 participants.

Category		Q1 N (%)	Q2 N (%)	Q3 N (%)	Q4 N (%)
Better	1	16 (23.2)	23 (33.3)	15 (21.7)	3 (4.3)
	2	30 (43.5)	27 (39.1)	21 (30.4)	10 (14.3)
	3	14 (20.3)	12 (17.4)	15 (21.7)	13 (18.6)
	4	5 (7.2)	4 (5.8)	8 (11.6)	6 (8.6)
	5	4 (5.8)	3 (4.3)	8 (11.6)	27 (38.6)
	6	0 (0.0)	0 (0.0)	2 (2.9)	11 (15.7)
Worse	7	0 (0.0)	0 (0.0)	0 (0.0)	0 (0.0)

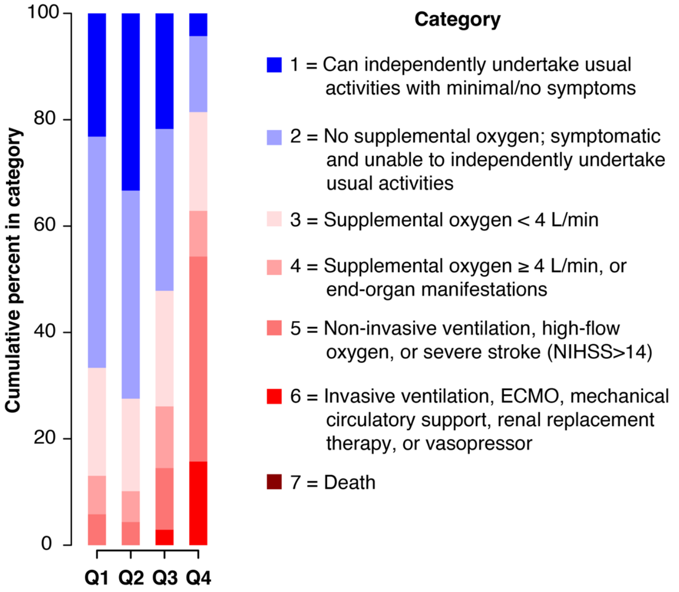

Supplemental Figure 2. Pulmonary ordinal outcome at day 5 by plasma sRAGE quartile.
